# Testing for IgA anti-tissue transglutaminase in routine clinical practice: Requesting behaviour in relation to prevalence of positive results

**DOI:** 10.1016/j.jtauto.2020.100045

**Published:** 2020-03-18

**Authors:** Maurits Damoiseaux, William van Doorn, Ellen van Lochem, Jan Damoiseaux

**Affiliations:** a Central Diagnostic Laboratory, Maastricht University Medical Center, Maastricht, the Netherlands; b Department of Microbiology and Immunology, Rijnstate Hospital, Arnhem, the Netherlands

**Keywords:** Coeliac disease, Serology, Diagnostic criteria

## Abstract

**Objective:**

Due to the high awareness of coeliac disease and improvement of serological tests, the number of requested laboratory tests has increased substantially over the years. In the current study we have evaluated the requesting behaviour of distinct clinical disciplines in relation to the prevalence of positive results and in the context of existing guidelines.

**Methods:**

Data were retrospectively extracted from the laboratory information system over a time-span of 5 years in a tertiary hospital and compared with the situation in a secondary hospital.

**Results:**

Data reveal that for initial testing (n=18,183) the percentage positive results for IgA anti-TTG is <2%. Paediatricians have a slightly higher percentage of seropositive results (2.4–4.0%). Early confirmation (<2 months) of positive results by IgA anti-endomysium antibodies in an independent sample is only performed in a minority of paediatric patients. The majority of positive patients, however, have follow-up measurements (<14 months) in order to examine compliance to a gluten-free diet. Interestingly, initial requests for paediatric patients reveal an equal distribution between boys and girls, while in adult patients there is a two times preponderance of requests in female patients, similar to the female/male ratio in patients with positive results, being either paediatric or adult patients.

**Conclusion:**

Although laboratory testing for coeliac disease may be primarily used to exclude the disease, it is evident that the percentage positive results for IgA anti-TTG is extremely low. This may indicate that the clinical manifestations that warrant testing, should be further specified in order to increase the pre-test probability. As the specific serology is important to bypass a biopsy in the diagnosis of coeliac disease according to the paediatric guideline, the confirmation in an independent sample needs to get more attention.

## Highlights

• The prevalence of IgA anti-TTG for diagnosis of coeliac disease is very low.
• The prevalence of IgA anti-TTG for diagnosis of paediatric patients is higher.
• The request for IgA anti-TTG occurs more often in females than in males (2:1).
• The request for IgA anti-TTG occurs as often in girls as in boys (1:1).
• Positive IgA anti-TTG are not consistently confirmed in an independent sample.

## 1 Introduction

Coeliac disease is a hypersensitivity reaction to gluten in genetically susceptible individuals resulting primarily in intestinal aberrations and, consequently, malabsorption. The diagnosis is based on a triad of clinical manifestations, histopathological findings and autoantibody serology [1,2]. Although coeliac disease typically presents with diarrhoea, in children resulting in growth retardation, patients may also present with extra-intestinal manifestations, for instance dermatitis herpetiformis or gluten ataxia. The discovery of tissue transglutaminase (TTG) as the antigen recognised by the autoantibodies in coeliac disease [3] has resulted in a more prominent role of serology in the diagnosis of coeliac disease. Indeed, in the 2012 ESPGHAN criteria for coeliac disease in children, histopathological confirmation of disease was considered no longer as mandatory [4]. More recently, this approach was reinforced in the 2020 ESPGHAN criteria [5]. Worldwide, it is well accepted that testing for IgA anti-TTG is the standard test for coeliac disease [1]. These tests have very good performance characteristics in terms of sensitivity and specificity [[6], [7], [8]]. Nevertheless, (inter)national guidelines still differ in the positioning of serology in the work-up for coeliac disease [4,5,[8], [9], [10]]. This includes the requirements for confirmation by serology and/or tissue biopsies.

The prevalence of coeliac disease is estimated between 0.5 and 1.0% of the population [11,12]. Coeliac disease is about 2–3 times more often diagnosed in females [2,13,14]. Although the prevalence seems to be increasing due to a true rise in incidence [1], it is also evident that over the years there is increased awareness and also the laboratory assays are more widely available. This, inevitably, has resulted in a strong increase in requests for laboratory tests for coeliac disease. The diverse and rather aspecific clinical manifestation of coeliac disease [1], however, hamper optimal selection of patients for serological testing. In this situation the test can be used for identifying the patients with coeliac disease or for exclusion of disease. Next, the test may also be used for screening of individuals at risk for development of coeliac disease, *i.e.*, first degree relatives, patients with autoimmune diseases associated with overlapping HLA-types, and patients with Down’s, Turner’s or Williams’ syndrome [[15], [16], [17], [18], [19], [20], [21], [22]]. Although it is anticipated that many patients with coeliac disease are not identified as such, referred to as the iceberg phenomenon [23], there is overall consensus that population screening is not warranted.

In the current retrospective study we investigated and compared the results for IgA anti-TTG serology in the patient population of the Maastricht University Medical Center (Maastricht, The Netherlands) and the Rijnstate Hospital (Arnhem, The Netherlands) for which coeliac disease testing was requested. This was analysed in a time-span of 5 years and requests for follow-up testing were initially excluded. First, we defined how often the initial test was positive. Second, we analysed whether there was a difference in requesting behaviour between clinical disciplines. Finally, we analysed the requesting behaviour for follow-up testing. The results are being discussed in the context of the existing (inter)national guidelines and recommendations for coeliac disease.

## 2 Materials & methods

### 2.1 Data selection

The datasets were extracted from the laboratory information system of the Maastricht University Medical Center (MUMC; Labosys, Philips, Eindhoven, The Netherlands) and of the Rijnstate Hospital (Arnhem, The Netherlands; GLIMS, MIPS Diagnostics Intelligence, Gent, Belgium). The MUMC dataset consisted of all IgA anti-TTG requests for the time period of January 1, 2011 to December 31, 2018. Besides the results for IgA anti-TTG, also other relevant data were extracted, including patient identification number, date of birth, sex, date of blood sampling, clinical department, and total IgA. As far as available, also results of IgA anti-endomysium antibodies (EMA) and IgG anti-TTG antibodies were extracted. To analyse the requesting policy for the initial diagnosis of coeliac disease only first entries in the study period of January 1, 2013 to December 31, 2017 had to be extracted. First, all test results in the period of interest that also had a request in the two years prior to our study period were excluded. Second, results of follow-up samples within the study period were excluded. Third, test results of referral hospitals and entries lacking results due to the absence of test material were excluded. Finally, data that deviated from the testing algorithm, *i.e.*, lacking total IgA results, were checked for being true first entries; if not, they were also excluded.

For follow-up analyses the same original MUMC dataset was used after removal of all patients that already had a test request for IgA anti-TTG before January 1, 2013. First, all patients with a single test request were excluded. Second, all patients with a first test request in 2018 were removed. Next, all patients from referral hospitals were removed. For final analyses only the first follow-up test results were included if requested within 14 months after the first request.

The Dataset from the Rijnstate Hospital consisted of all requests combining both IgA anti-TTG and total IgA for the time period of January 1, 2013 to December 31, 2017. Since the Rijnstate Hospital intends to measure total IgA only at the first request for IgA anti-TTG, this dataset almost fulfilled our criteria for analysing the requesting policy for the initial diagnosis of coeliac disease. Based on duplicate patient identification numbers, 32 entries had to be excluded. Also, 17 samples that lacked IgA anti-TTG results were excluded. Additional data extracted from the laboratory information system of the Rijnstate Hospital were similar as for the MUMC, except that for all requests IgA EMA and occasionally also IgG EMA was determined.

For follow-up analyses an additional data extraction from the laboratory information system was obtained, including all follow-up results from January 1, 2013 to December 31, 2019. Next, only the first follow-up results were included and linked to the dataset for the diagnostic requesting policy. For final analyses only the first follow-up test results were included if requested within 14 months after the first request.

Only for the MUMC cohort clinical data were extracted from the patient charts based on the patient identification number. This involved all patients being originally positive for IgA anti-TTG and a random selection of the patients being originally negative for IgA anti-TTG, but with a follow-up measurement within 14 months.

### 2.2 Testing algorithm for coeliac disease

In the MUMC, patients suspected for having coeliac disease were tested for IgA anti-TTG (EliA Celikey IgA, Thermo Fisher Diagnostics, Freiburg, Germany) in combination with total IgA by nephelometry (BN Prospect, Siemens, Den Haag, The Netherlands). Patients were considered positive if IgA anti-TTG was ≥10 U/mL. Positive results were confirmed by IgA EMA by indirect immunofluorescence (IIF) on monkey oesophagus tissue (SciMedx, Dover, NJ). An initial request for IgA EMA was allowed for clinicians from the Maastricht University Medical Center, but not for general practitioners. Although IgA deficiency was defined as a total IgA <0.07 ​g/L, all samples with results of total IgA <0.20 ​g/L were tested for IgG anti-TTG (EliA Celikey IgG, Thermo Fisher Diagnostics). All assays were performed according to the instructions of the manufacturers. In case of follow-up it is advised to request only for IgA anti-TTG, or IgG anti-TTG in case of IgA deficiency, but additional test requests are rewarded.

In the Rijnstate Hospital a slightly different algorithm is implemented. At the initial request testing for IgA EMA is always included and in case of low or deficient IgA, *i.e.*, total IgA <0.20 ​g/L, IgG EMA are being tested for. If IgG EMA are positive, confirmation by IgG anti-TTG is not consistently performed. For IgA and IgG anti-TTG the Celikey tests of Thermo Fisher Diagnostics were used. Comparable to the MUMC setting, Celikey tests of Thermo Fisher Diagnostics were used for IgA and IgG anti-TTG. For total IgA nephelometry was used (BN II, Siemens, Den Haag, The Netherlands). IgA EMA tests were performed using IIF tests (Mosaic: monkey intestine/oesophagus; Euroimmun AG, Lübeck, Germany).

### 2.3 Statistics

Statistical analyses were performed in SPSS (version 25.0; SPSS Inc., Chicago, IL) and GraphPad Prism (version 5; GraphPad; San Diego, CA). Descriptive statistics were computed for all variables in the total cohort as well as separately in all distinct clinical disciplines. The variables were plotted in a frequency histogram to confirm a normal distribution of the variable within the total cohort. Statistical differences were confirmed by performing a one-way ANOVA followed either by a post-hoc Pearson’s chi-square test regarding categorical data or an independent, two-sided *t*-test for continuous data. Statistical significance was set at a=0.05.

## 3 Results

### 3.1 Patient characteristics

In the retrospective cohort of 2013–2017 the sera of 7055 patients were tested in the MUMC for the diagnostic work-up of coeliac disease. The patient characteristics and test results are presented in Table 1A. The percentage females of the total cohort was 63.6% and the mean age was 38 years (±21.4). Total IgA was on average 1.95 ​g/L (±1.11) with a prevalence of 0.48% IgA deficiency (n=34). Only 109 patients (1.55%) were positive for IgA anti-TTG. All samples with a level >35 U/mL (n=70) were confirmed positive by IgA EMA. The samples in the range 10–35 U/mL (n=39) were differentially confirmed: 16 samples were negative, 15 samples weak positive, and 8 samples positive for IgA EMA. Only 74 (67.9%) out of the 109 patients positive for IgA anti-TTG had a final diagnosis of coeliac disease. In 16 patients (14.7%) the diagnosis was uncertain and in 19 patients (17.4%) the diagnosis was rejected. Within the cohort of 34 patients with an IgA deficiency and 34 patients with low IgA levels (0.07–0.20 ​g/L), only 1 serum from an IgA deficient patient was positive for IgG anti-TTG (107 U/mL).

**Table 1A:** Patient characteristics and test results of the MUMC cohort differentiated for clinical discipline of requesting.

MUMC	Total	GP	PAED	GE	INT	NEU	Other
***N***	7055	1552	1241	1574	1016	1455	217
**Females** ***N* (%)**	4488 (63.6)	1034 (66.7)	647 (52.1)	1040 (66.1)	722 (71.1)	917 (58.3)	128 (59.0)
**Age** **Mean (SD)**	38 (21.4)	35 (19.2)	9 (7.1)	45 (17.2)	41 (17.9)	52 (14.4)	47 (21.7)
**Total IgA** **Mean (SD)**	1.95 (1.11)	1.93 (0.96)	1.16 (0.78)	2.27 (1.23)	2.16 (1.16)	2.13 (0.96)	2.25 (1.28)
**IgA deficiency** ***N* (%)**	34 (0.48)	6 (0.39)	7 (0.56)	6 (0.38)	9 (0.89)	3 (0.21)	3 (1.38)
**IgA anti-TTG +** ***N* (%)**	109 (1.55)	21 (1.35)	50 (4.03)	13 (0.83)	12 (1.18)	5 (0.34)	8 (3.69)
**Females** **IgA anti-TTG +** ***N* (%)**	68 (62.4)	15 (71.4)	33 (66.0)	9 (69.2)	6 (50.0)	1 (20.0)	4 (50.0)

Abbreviations: GE, gastroenterology; GP, general practitioners; INT, internal medicine; MUMC, Maastricht University Medical Center; NEU, neurology; PAED, paediatrics; TTG, tissue transglutaminase.

In the Rijnstate Hospital a total of 11,128 sera, collected from new patients, was tested for coeliac disease during the same study period as above. The patient characteristics and test results are presented in Table 1B. The percentage females was 63.1% and the mean age was 32 years (±22.1). Total IgA was on average 1.83 ​g/L (±1.17) with a prevalence of 0.57% IgA deficiency (n=63). Only 213 patients (1.91%) were positive for IgA anti-TTG. All samples with a level >35 U/mL (n=146) were confirmed by IgA EMA testing. The samples in the range 10–35 U/mL (n=68) were IgA EMA negative (n=12), weak positive (n=16), or positive (n=39); for 1 sample the IgA EMA result was lacking. Additionally, 9 of the 63 sera from IgA deficient patients were positive for IgG EMA; 6 of these sera were tested for IgG anti-TTG and all tested positive. The cohort of patients (n=82) with low IgA levels (0.07–0.20 ​g/L) were only partially (n=52) tested for IgG EMA and all tested negative.

**Table 1B:** Patient characteristics and test results of the Rijnstate cohort differentiated for clinical discipline of requesting.

Rijnstate	Total	GP	PAED	GE	INT	Other
***N***	11,128	3212	2641	3156	1852	267
**Females** ***N* (%)**	7024 (63.1)	2079 (64.7)	1416 (53.6)	2054 (65.1)	1275 (68.8)	200 (74.9)
**Age** **Mean (SD)**	32 (22.1)	30 (19.4)	8 (5.4)	45 (17.4)	47 (17.9)	47 (19.0)
**Total IgA** **Mean (SD)**	1.83 (1.17)	1.80 (1.04)	1.08 (0.67)	2.22 (1.19)	2.27 (1.33)	2.25 (1.19)
**IgA deficiency** ***N* (%)**	63 (0.57)	14 (0.44)	26 (0.98)	14 (0.44)	7 (0.38)	2 (0.75)
**IgA anti-TTG +** ***N* (%)**	213 (1.91)	45 (1.40)	64 (2.42)	71 (2.25)	29 (1.57)	4 (1.50)
**Females** **IgA anti-TTG +** ***N* (%)**	141 (66.2)	26 (57.8)	41 (64.1)	50 (70.4)	20 (69.0)	4 (100.0)

Abbreviations: GE, gastroenterology; GP, general practitioners; INT, internal medicine; NEU, neurology; PAED, paediatrics; TTG, tissue transglutaminase.

### 3.2 Differences in requesting behaviour between clinical disciplines

**Fig. 1:**
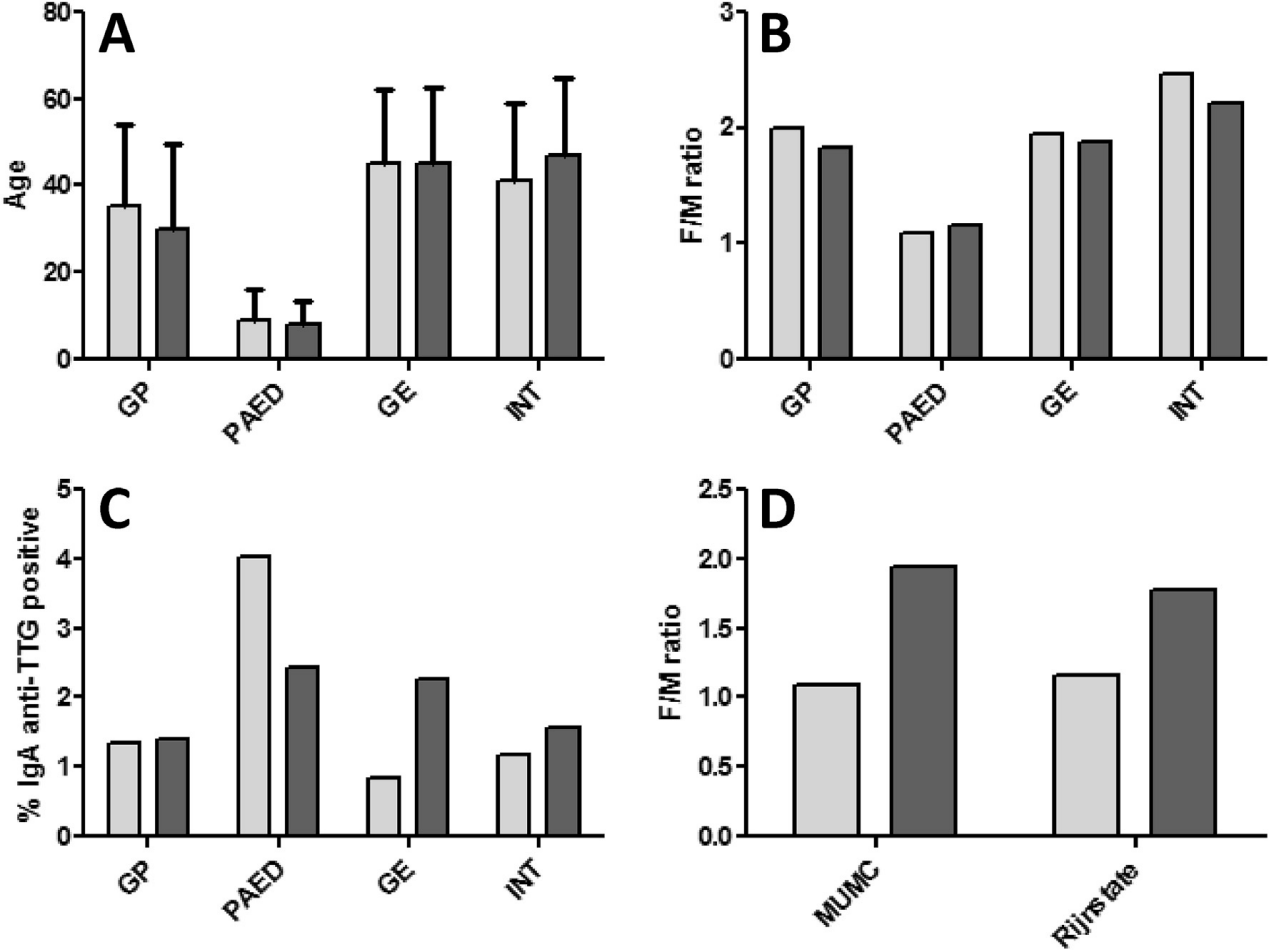
Requesting characteristics for IgA anti-TTG. A: age distribution (mean ​± ​SD) according to distinct clinical disciplines in the MUMC (light) and the Rijnstate Hospital (dark); B: F/M ratio according to distinct clinical disciplines in the MUMC (light) and the Rijnstate Hospital (dark); C: frequencies (%) of IgA anti-TTG positive results according to distinct clinical disciplines in the MUMC (light) and the Rijnstate Hospital (dark); D: F/M ratio in the paediatric population for which IgA anti-TTG testing was requested (light) and for which IgA anti-TTG testing was positive (dark) in the MUMC and the Rijnstate Hospital, respectively. Abbreviations: GE, gastroenterology; GP, general practitioners; INT, internal medicine; PAED, paediatrics.

The major clinical disciplines that request testing for coeliac disease in the MUMC were general practitioners, paediatricians, gastroenterologists, specialists in internal medicine, and neurologists. Test requests by neurologists might be unexpected, but this is explained by the MUMC being a referral center for small-fiber neuropathy; serological testing for coeliac disease is included in the local protocol. There are a number of apparent differences between the distinct clinical disciplines (Table 1A and Fig. 1). First, the percentage females is substantially lower in the paediatric sample (52.1%) as compared to the other disciplines (58.3–71.1%; all comparisons p ​< ​0.001) (Fig. 1B). Second, the paediatric sample also had lower total levels of IgA (1.16 ​g/L [±1.17]) as compared to the other clinical disciplines (1.93–2.27 ​g/L; all comparisons p ​< ​0.001). Also IgA levels in the general practitioner sample tended to be lower compared to the other distinct adult cohorts due to the inclusion of children (all comparisons p ​< ​0.001). Finally, the percentage positive results for IgA anti-TTG was significantly higher in the paediatric sample (4.03% *vs* 0.34–1.35%; all comparisons p ​< ​0.001) (Fig. 1C). Interestingly, while the percentage females in the total paediatric sample was 52.1% (Fig. 1B), this was 66.0% in the children with a positive IgA anti-TTG, *i.e.*, similar to the percentage females in the total cohort of positive patients (62.4%; p ​> ​0.05) (Fig. 1D). This percentage remained similar in the paediatric subcohort in which the positive IgA anti-TTG results was confirmed by IgA EMA (63.8%; data not shown).

The major clinical disciplines that request tests for coeliac disease in the Rijnstate Hospital were the same as for the MUMC with exception of the neurology department. Also, in this cohort there are a few perceptible differences between the distinct clinical disciplines (Table 1B and Fig. 1). First, the percentage females is substantially lower in the paediatric sample (53.6%) as compared to the other disciplines (64.7–68.8%; all comparisons p ​< ​0.001) (Fig. 1B). Second, total IgA levels (1.08 ​g/L [±0.67]) were also significantly lower in the Rijnstate Hospital in the paediatric sample (all comparisons p ​< ​0.001). To a lesser extent, this was also apparent in the general practitioner sample (1.80 ​g/L [±1.04]). Finally, as compared to the general practitioner sample (1.40%), the percentage positive results for IgA anti-TTG was higher in both the paediatric sample (2.42%; p ​< ​0.01), as well as the gastroenterology sample (2.25%; p ​< ​0.05) (Fig. 1C). As observed in the MUMC, the percentage females within the children with a positive IgA anti-TTG was similar to percentage females in the total positive cohort (64.1% *vs* 66.2%; all comparisons p ​> ​0.05) (Fig. 1D).

### 3.3 Comparison of the MUMC versus the Rijnstate Hospital cohort

When concentrating on the four major clinical disciplines, it is apparent that the proportion of requests in the MUMC and the Rijnstate Hospital is quite similar for the general practitioners (29.6% *vs* 28.8%), paediatrics (24.3% *vs* 23.1%), gastroenterologists (29.1% *vs* 29.2%), and internal medicine (17.1% *vs* 18.9%). There are some clear similarities between both hospitals: percentage females (63.6% *vs* 63.1%), total IgA (1.95 ​g/L *vs* 1.83 ​g/L) (Table 1A, Table 1B), the percentage IgA deficiency (0.48% *vs* 0.57%), and percentage positive IgA anti-TTG results (1.55% *vs* 1.91%). Furthermore, both paediatric samples consistently deviated from the other clinical disciplines, in terms of a lower percentage females, lower age and total IgA, but higher percentage positive IgA anti-TTG results (Fig. 1). However, there are also some differences. In the total cohort, the mean age in the MUMC (38 ​yrs) is higher than in the Rijnstate Hospital (32 ​yrs). This is attributed to the higher age (52 ​yrs) of the large content of neurology patients in the MUMC (20.6%). Furthermore, the percentage children within the sample of the general practitioners was higher in the Rijnstate Hospital (31.1%) as compared to the MUMC (16.9%). With respect to the major outcome of our study, *i.e.*, the percentage positive results, it is apparent that the MUMC paediatric sample revealed a higher percentage of positive results than the Rijnstate Hospital paediatric sample (4.03% *vs* 2.42%; p ​< ​0.01). In the Rijnstate Hospital, on the other hand, the gastroenterology sample had a higher percentage of positive results (2.25% *vs* 0.83%; p ​< ​0.01).

### 3.4 Requesting pattern for follow-up

The retrospective cohort of 2013–2017 consisted of 7055 new patients being tested for coeliac disease in the MUMC. As already mentioned, 109 patients tested positive for IgA anti-TTG. Only for 71 patients (65.1%) follow-up serology was available. Some patients (n=7) were referred to other hospitals for follow-up. First, follow-up testing was analysed in the context of the diagnostic work-up. The ESPGHAN guideline requires confirmation of a positive result for IgA anti-TTG by IgA EMA in an independent sample [5]. For this we defined a window of 2 months for confirmation. In the paediatric sample (n=50) follow-up data within 2 months was available for 14 patients (28.0%). In the non-paediatric sample (n=59) serologic confirmation occurred in only 2 patients (3.4%). Second, follow-up testing in patients with a final diagnosis of coeliac disease (n=74) is indicated in order to evaluate the effect of a gluten free diet. Follow-up within 14 months occurred in 62 patients (83.8%); 3 patients were referred to another hospital. Finally, only 196 patients (2.8%) were re-analysed after a negative first result for IgA anti-TTG. This might be relevant in terms of repeated testing in patients at risk for coeliac disease. A random selection of 40 patients indicated that 34 patients (85%) only had a single follow-up, while 6 patients (15%) had multiple follow-up analyses. Only 3 of the latter group underwent repeated screening because of increased risk for coeliac disease due to type 1 diabetes mellitus.

In the Rijnstate Hospital, for 169 of the 213 patients (79.3%) positive for IgA anti-TTG follow-up serology within a time-span of 14 months was available. With respect to confirmation within 2 months, paediatricians requested a confirmation test in 24 out of 64 (37.5%) IgA anti-TTG positive patients. In the non-paediatric sample (n=145), only 9 (6.2%) confirmation tests were requested within 2 months. Finally, only 420 patients (3.8%) were re-analysed after a negative first result for IgA anti-TTG.

## 4 Discussion

The current retrospective study was aimed to investigate the requesting behaviour of distinct clinical disciplines for serological tests for coeliac disease. This investigation further focussed on the prevalence of positive results, sex differences in the respective patient subsets, and serological follow-up of patients originally tested positive or negative in the context of existing guidelines and recommendations.

With respect to the prevalence of positive results, it was surprising that the percentage of positive IgA anti-TTG results within the MUMC study population (1.6%) is only slightly higher than the prevalence of coeliac disease in the general population (0.5–1.0%) [11,12]. Importantly, this finding was confirmed in an independent cohort of the Rijnstate Hospital (1.9%). These data suggest that the requesting criteria of serologic testing for coeliac disease are rather aspecific. In this respect, it is interesting that paediatricians of the MUMC (4.0%) have significantly higher positive rates than clinicians in other disciplines. Again, this finding was confirmed in the Rijnstate Hospital (2.4%) participating in our study. Possibly, the clinical manifestations leading to testing for coeliac disease in children, like growth retardation, are more specific than the ones used in the adult population. Furthermore, in the Rijnstate Hospital also the gastroenterologists (2.3%) had significantly higher positive rates, while in the MUMC the neurologists (0.3%) had very low positive rates. These findings were not confirmed in the other participating hospital. The difference could be explained by the two hospitals being expertise centers for certain diseases. Indeed, the department of gastroenterology of the Rijnstate Hospital is a regional referral center for coeliac disease, while the MUMC is specialized in small-fiber neuropathy [24,25]. The latter results in a rather broad autoantibody screening. Our results, however, do not support any relevance of such screening for coeliac disease. The indications of the National Institute for Health and Care Excellence for indications that should prompt testing for coeliac disease, therefore, should be reconsidered, in particular with respect to the item of “unexplained neurological symptoms” [26]. Our conclusion is that the gating strategy for coeliac disease as based on clinical manifestation is rather aspecific and is focussed on case finding. This is in line with the Dutch guidelines for general practitioners [9] in which testing for coeliac disease is recommended to be considered in cases of unexplained chronic intestinal complaints, anaemia, weight loss or growth retardation in children. In addition, there is a national guideline for coeliac disease that recommends awareness for the disease in patients with type 1 diabetes mellitus, osteoporosis, thyroid disease, microscopic colitis, autoimmune diseases in general, elevated levels of transaminases or iron deficiency anaemia [10]. In the same consensus it is anticipated that the prevalence of coeliac disease in adult patient suspected for this disease is 12–54%, while in children this is 4.6–17%. Evidently, this is much higher than observed in our current study. Internationally, both the guidelines for children as well as adults provide further details about the symptoms and signs suggesting coeliac disease and, consequently, should be tested by serology [5,8]. Unfortunately, our dataset does not contain information about the clinical manifestations that have resulted in a test request. Alternatively, testing for coeliac disease may also be requested in order to exclude the disease. Since the sensitivity of the IgA anti-TTG test is very high, this is accompanied by a high negative predictive value and, as such, very useful to exclude the disease by a single blood test. This situation is in line with our data showing that by far the majority of negative results are not repeated.

Coeliac disease is diagnosed more frequently in women with a female-to-male ratio ranging from 2:1 to 3:1 [2,13,14]. This is often explained by the fact that coeliac disease is considered an autoimmune disease. Although this can be disputed [27], it is to be anticipated that the observed female-to-male ratio is also reflected in the requesting behaviour. Both in the MUMC and the Rijnstate Hospital, indeed, a female-to-male ratio of ~2:1 was observed in the patient population that was tested for IgA anti-TTG. The same ratio was observed in the patients that tested positive. However, paediatricians appeared to test their patients in a ~1:1 ratio. This might be explained by a difference in the female-to-male ratio of the clinical manifestations prompting for testing for coeliac disease between children and adults. Interestingly, the female-to-male ratio in the children that test positive for IgA anti-TTG again is ~2:1.

Repeated testing for IgA anti-TTG is important for follow-up of patients with coeliac disease for which a gluten free diet has been installed. Overall, clinicians do not follow a standard scheme for monitoring. As expected, for the vast majority of patients diagnosed with coeliac disease, follow-up testing was requested. Another reason for follow-up testing is confirmation of positive results in an independent blood sample. This is part of the guideline for children [5], but not for adults [8]. Our data show that confirmation within 2 months is more prevalent in the paediatric cohort as compared to the adult cohort, but in the paediatric cohort positive test results are not confirmed in a substantial number of patients. Finally, follow-up testing may be requested for patients at risk for developing coeliac disease. Since patients may develop positive serology and coeliac disease over time, it is advised to monitor these patients every year, unless a predisposing HLA haplotype has been excluded [8]. Although it has not been established how many patients in our study were at risk for coeliac disease, the percentage follow-up requests after a negative result for IgA anti-TTG was very low (2.8–3.8%). Since it can be expected that the majority of first requests are based on clinical manifestations and not on being at risk for coeliac disease, these data indicate that clinicians consider a negative test result as very reliable.

The reason behind a serological test request is an important part of the requesting behaviour. Tests could either be requested to confirm or rule out a certain disease. Due to the retrospective approach, the reasons for requesting serology were not available in the current study. On top of that, the data consisting of initial requests may actually concern follow-up test, because we only excluded patients that had requests for IgA anti-TTG up to two years prior to the study period. Additionally, patients may have been referred to our hospitals after testing positive in another clinical setting. A second draw-back of our study is that a positive test result for IgA anti-TTG was considered a diagnostic marker for coeliac disease. However, the positive predictive value appeared to be 67.9–82.6%, depending on in- or exclusion of patients for which the diagnosis was not yet definite. Altogether, a prospective study design may provide additional information about the requesting behaviour. Next to that, the study data is obtained in two hospitals based in the Netherlands. Therefore, possible international differences are not disclosed, which also may reveal supplementary conclusions for the requesting behaviour of laboratory tests for coeliac disease.

In conclusion, our data show that the frequency of positive IgA anti-TTG is unexpectedly low. This is probably attributed to the fact that the clinical manifestations in the disease criteria for coeliac disease are not very specific. Apparently, testing is primarily used to exclude the disease. This is in line with the finding that there is hardly any follow-up of negative results. Furthermore, it is interesting that paediatricians, in contrast to other clinical disciplines, have an equal distribution between the sexes for which IgA anti-TTG testing is requested. Nevertheless, the paediatric patients with a positive result have the expected female preponderance. Possibly, the clinical manifestations in children are more equally distributed between boys and girls. Finally, confirmation of a positive result in an independent follow-up sample, as directed by the ESPGHAN guideline [4,5], is requested in less than half of the paediatric patients. Obviously, these data should be confirmed in an international prospective study that may give further guidance toward appropriate clinical manifestations that warrant testing for coeliac disease.

## Funding

This research did not receive any specific grant from funding agencies in the public, commercial, or not-for-profit sectors.

## Contributors

JD designed the study, JD and EvL advised on collection of laboratory data, MD analysed the data files, WvD supported in statistical analyses, MD and JD drafted the manuscript, MD, WvD, EvL and JD edited and approved the final version of the manuscript.

## Patient consent

Retrospective study using routine diagnostic data in an anonymous way.

## Ethics committee

Since data were analysed in an anonymous way in a retrospective study design, separate ethical approval for this study was not necessary according to Dutch ethical guidelines.

## Declaration of competing interest

The authors declare no conflicting interests.
